# Association between defecation status and the habit of eating vinegar-based dishes in community-dwelling Japanese individuals: a cross-sectional study

**DOI:** 10.1038/s41598-025-95618-2

**Published:** 2025-03-28

**Authors:** Yuto Aoki, Shin Kawasoe, Takuro Kubozono, Joto Yoshimoto, Mikiya Kishi, Hiroaki Kanouchi, Satoko Suzuki, Mitsuru Ohishi

**Affiliations:** 1 Central Research Institute, Mizkan Holdings Co., Ltd, Handa-shi, Aichi Japan; 2 Department of Cardiovascular Medicine and Hypertension, Graduate School of Medical and Dental Sciences, Kagoshima University, Kagoshima-shi, Kagoshima Japan; 3 Department of Clinical Nutrition, Osaka Metropolitan University, Osaka-shi, Osaka Japan; 4 Department of Nutrition Management, Imakiire General Hospital, Kagoshima-shi, Kagoshima Japan

**Keywords:** Constipation, Dietary fiber, Vinegar-based foods, Eating habits, Defecation improvement, Incomplete evacuation, Nutrition, Quality of life

## Abstract

Vinegar intake reportedly has an antihypertensive effect and reduces visceral fat. Nonetheless, studies on the form of vinegar intake and its effect on defecation are scarce. This cross-sectional study aimed to investigate the association between the frequency of vinegar-based dish intake and defecation status using data from the Tarumizu cohort study. The participants (*n* = 1024, 634 women) responded to a health check survey in 2019 using a brief-type self-administered diet history questionnaire. The association between the frequency of vinegar-based dish intake and defecation status was examined using a multivariate logistic regression analysis. Considering confounding factors influencing the defecation status such as sex, age, dietary fiber intake, and medication history, individuals with a habit of eating vinegar-based dishes, such as “sour main dishes” (odds ratio [OR]: 1.38; *p* = 0.039), “sunomono” (OR: 1.49; *p* = 0.035), and “salad with sour dressing” (OR: 1.41; *p* = 0.049), had a significantly higher defecation frequency. No significant association was observed between the habit of eating vinegar-based dishes and the time required for defecation or straining during defecation. Our study showed that the habit of eating vinegar-based dishes was positively associated with defecation status. Our findings may suggest a novel approach for defecation improvement in people with defecation problems.

## Introduction

Excretion is an indispensable activity of daily living. An individual in a state of constipation, who cannot excrete feces satisfactorily, has a sensation of incomplete evacuation and experiences abdominal discomfort, leading to a decline in mental and physical quality of life^1^. Moreover, constipation not only leads to a decline in quality of life but is also a factor reportedly associated with an increased risk of cardiovascular disease and stroke^2^; hence, constipation has a negative impact on life prognosis as well. The prevalence of constipation is as high as 10–20% overall^3–7^ and increases even more in older people^5,7,8^. Studies focusing exclusively on Japanese populations have reported similar findings, with the prevalence of constipation ranging from approximately 3.5–28%^9–11^. According to the Comprehensive Survey of Living Conditions in Japan in 2019, the overall prevalence of constipation was 34.8 per 1,000 persons, while in the older population, it was higher at 68.5 per 1,000 persons, consistent with global trends^11^. The primary treatment plan for constipation is to improve lifestyle habits, such as diets high in dietary fiber and an increase in physical activity or fluid intake; in case of no improvement, medication customized to the symptoms is selected^12^. Exercise and increased fluid intake are beneficial for treating constipation, but there are no data to support that increasing the amount of exercise and fluid intake improves chronic constipation except in cases of dehydration^12–15^. A meta-analysis evaluating the relationship between dietary fiber intake and constipation has reported that dietary fiber intake clearly increases stool frequency in patients with constipation^16^. However, despite the recognized importance of dietary fiber, some challenges still need to be addressed, such as the fact that dietary fiber intake is decreasing annually in Japan^17^, and only approximately 5% of the population in the United States consumes the recommended amount of dietary fiber^18^. Considering such a background, a new approach to improving eating habits that can improve defecation is eagerly awaited.

The primary regulator of gastrointestinal motility is the enteric nervous system, with various factors such as the gut microbiota, central nervous system, and immune system also interacting to regulate gastrointestinal motility^19^. A disruption in the balance of any of the gastrointestinal motility regulators may lead to abnormal intestinal activity, causing symptoms such as constipation^19^. The gut microbiota interacts with various factors, including the enteric nervous system, through metabolites generated by gut fermentation, playing an important role in regulating gastrointestinal motility^19^. The gut microbiota produces various metabolites with a wide range of biological activities. Among these, short-chain fatty acids are a group of intestinal microbiota metabolites that have been widely studied and have attracted attention^20^. Short-chain fatty acids are fatty acids with less than six carbon atoms (C). Common short-chain fatty acids produced by gut fermentation are acetic acid (C2), propionic acid (C3), and butyric acid (C4), which are present in molar ratios of 60:20:20, respectively^21^. Various studies have elucidated the diverse physiological effects of short-chain fatty acids produced in the intestine. Previous studies have also verified the relationship between short-chain fatty acids and defecation; in animal experiments using mice, it was reported that increasing acetic acid concentrations in the large intestine improved defecation frequency or caused serotonin-mediated stimulation of peristalsis^22–24^.

Vinegar, which contains acetic acid as the main constituent, is a seasoning with a long history of use since ancient times. Various health benefits of vinegar intake have been reported. For example, the antihypertensive effect through the ingestion of vinegar-based drinks^25^ and visceral fat reduction^26^ have been demonstrated in animal experiments and clinical trials. However, to the best of our knowledge, only few studies have examined the effect of oral vinegar ingestion on improving defecation. Moreover, while most existing interventional studies have reported on the health benefits of vinegar in the form of vinegar drinks, considering dietary habits, non-beverage forms of intake are also interesting research topics. Therefore, using a nutritional epidemiological approach, we performed an analysis focusing on people who had a habit of eating vinegar-based dishes and their defecation status.

A health survey incorporating the “frequency of eating dishes containing vinegar” as a questionnaire item has been previously conducted in Tarumizu City, Kagoshima Prefecture^27–29^. The questionnaire items for the “frequency of eating dishes containing vinegar” were classified into staple food, main dish, and side dish, and the side dishes were further classified into “sunomono,” which means “dressed with vinegar” in Japanese, and “salad with sour dressing.” By analyzing the Tarumizu cohort data, a negative correlation between vinegar intake habits and blood pressure levels has been reported^27^; however, the association between vinegar intake habits and any other physical conditions has not been examined. The current study aimed to investigate the association between the habit of consuming vinegar-based dishes and defecation status and to consider the form of vinegar intake that can improve defecation by analyzing the Tarumizu cohort data.

## Methods

### Study design and population

This cross-sectional study used data from the Tarumizu cohort study, conducted in 2019^28,29^. The Tarumizu cohort study is a health check survey of men and women aged ≥ 40 years living in Tarumizu City. Participants applied through newspaper posting and community events, and 1024 individuals were registered in the Tarumizu cohort study in 2019. All participants were included in the analysis. Informed consent was obtained from all participants before inclusion in the study, and the study protocol was approved by the Ethics Committee of the Faculty of Medicine, Kagoshima University (approval number: 220135; approval date: October 24, 2022). All methods were performed in accordance with the relevant guidelines and regulations. The Strengthening the Reporting of Observational Studies in Epidemiology-Nutritional Epidemiology (STROBE-nut) checklist was used for the reporting of this study.

### Diet history questionnaire

We utilized the brief-type self-administered diet history questionnaire (BDHQ)^30,31^ designed for people living in Japan to evaluate dietary behavior indicators, such as nutrient content and food intake, based on dietary intake habits in the last 1 month. Data on the frequency of vinegar-based dish intake were collected through interviews with the participants in the Tarumizu cohort study health check survey^27^. For the frequency of intake of “sour main dishes,” “sunomono,” “pickles,” and “salad with sour dressing” the participants selected one of seven choices: “2 times or more/day,” “1 time/day,” “4–6 times/week,” “2–3 times/week,” “1 time/week,” “less than 1 time/week,” or “not at all in the last 1 month.” For the frequency of “sushi” intake, the participants selected one of six choices: “5 times or more/month,” “4 times/month,” “3 times/month,” “2 times/month,” “1 time/month,” or “not at all in the last 1 month.” The responses on “sushi” intake frequency were excluded from the analysis because compared with other items in the questionnaire, the frequency was set to be quite low. The responses on vinegar-based dish intake frequency were divided into two groups, with 1 time or more/week as the “with group” and with less than 1 time/week as the “without group.”

### Defecation status

In the Tarumizu cohort study health check survey, participants were asked for data on three items regarding the defecation status: “Do you have daily defecation?” “How long does it take to defecate?” “Do you strain during defecation?” The participants responded by selecting from two choices of “daily” or “not daily” for “Do you have daily defecation?” three choices of “less than 5 min,” “5–10 min,” and “more than 10 min” for “How long does it take to defecate?” and four choices of “daily,” “frequently,” “sometimes,” or “rarely” for “Do you strain during defecation?”

In our evaluation, the responses to the question “How long does it take to defecate?” were divided into two groups: the “less than 5 min” group and the “5 min or more” group. For “Do you strain during defecation?” the responses were divided into two groups, with “rarely” as the “without straining” group and “sometimes,” “frequently,” or “daily” as the “with straining” group.

### Covariates

In this study, age (years), sex (male/female), total dietary fiber intake (g/day), and history of using medications (stomachics, probiotics, laxatives) that may affect defecation status were treated as covariates. Participants with a medication history were marked as “Yes,” whereas those without a medication history were marked as “No.” The total dietary fiber intake (g/day) was calculated using the BDHQ data.

### Statistical analysis

All analyses were performed using JMP version 17.0 (SAS Institute Inc.), with a p-value of < 0.05 being considered statistically significant. The chi-squared test for categorical variables and Welch’s t-test for continuous variables were used to compare the two groups created based on responses regarding the defecation status. A multivariate logistic regression analysis was conducted to investigate the association between vinegar-based dish intake habits (1 time or more/week) and defecation status, with defecation status as the dependent variable. The first logistic regression model (Model 1) was adjusted for age and sex as covariates. The second logistic regression model (Model 2) was adjusted for age, sex, dietary fiber intake (g/day), and medication history as covariates.

## Results

### Participant characteristics

Tables 1 and 2 summarize the participant information and present the results of the comparison between the two groups created based on responses regarding respective items in the defecation questionnaire.

**Table 1:** Participant information on the habits of eating vinegar-based dishes and defecation status.

	Total (*n* = 1024)	Group with daily defecation (*n* = 799)	Group without daily defecation (*n* = 225)	*p*	Less than 5 min Group (*n* = 806)	5 min or more Group (*n* = 218)	*p*	Group without straining (*n* = 537)	Group with straining (*n* = 487)	*p*
Sour main dishes n (%)				0.007			0.562			0.452
≥ 1 time/week	572 (55.9)	464 (58.1)	108 (48.0)		454 (56.3)	118 (54.1)		294 (54.7)	278 (57.1)	
< 1 time/week	452 (44.1)	335 (41.9)	117 (52.0)		352 (43.7)	100 (45.9)		243 (45.3)	209 (42.9)	
Sunomono n (%)				0.020			0.059			0.608
≥ 1 time/week	804 (78.5)	640 (80.1)	164 (72.9)		643 (79.8)	161 (73.9)		425 (79.1)	379 (77.8)	
< 1 time/week	220 (21.5)	159 (19.9)	61 (27.1)		163 (20.2)	57 (26.1)		112 (20.9)	108 (22.2)	
Pickles n (%)				0.270			0.894			0.778
≥ 1 time/week	502 (49.0)	399 (49.9)	103 (45.8)		396 (49.1)	106 (48.6)		261 (48.6)	241 (49.5)	
< 1 time/week	522 (51.0)	400 (50.1)	122 (54.2)		410 (50.9)	112 (51.4)		276 (51.4)	246 (50.5)	
Salad with sour dressing n (%)				0.040			0.909			0.532
≥ 1 time/week	764 (74.6)	608 (76.1)	156 (69.3)		602 (74.7)	162 (74.3)		405 (75.4)	359 (73.7)	
< 1 time/week	260 (25.4)	191 (23.9)	69 (30.7)		204 (25.3)	56 (25.7)		132 (24.6)	128 (26.3)	

Data were expressed as number (%).

Proportions for categorical data were compared using chi-square test.

**Table 2:** Participant information on characteristics, medication history, TDF intake, and defecation status.

	Total (*n* = 1024)	Group with daily defecation (*n* = 799)	Group without daily defecation (*n* = 225)	*p*	Less than 5 min Group (*n* = 806)	5 min or more Group (*n* = 218)	*p*	Group without straining (*n* = 537)	Group with straining (*n* = 487)	*p*
Age (years), Mean (SD)	68 (11)	68 (11)	68 (12)	0.954	68 (11)	69 (11)	0.559	68 (11)	69 (11)	0.032
Sex n (%)				< 0.001			< 0.001			0.401
Male	390 (38.1)	336 (42.1)	54 (24.0)		280 (34.7)	110 (50.5)		198 (36.9)	192 (39.4)	
Female	634 (61.9)	463 (57.9)	171 (76.0)		526 (65.3)	108 (49.5)		339 (63.1)	295 (60.6)	
Stomachics n (%)				0.094			0.824			0.498
Yes	74 (7.2)	52 (6.5)	22 (9.8)		59 (7.3)	15 (6.9)		36 (6.7)	38 (7.8)	
No	950 (92.8)	747 (93.5)	203 (90.2)		747 (92.7)	203 (93.1)		501 (93.3)	449 (92.2)	
Laxatives n (%)				0.260			0.868			0.050
Yes	22 (2.1)	15 (1.9)	7 (3.1)		17 (2.1)	5 (2.3)		7 (1.3)	15 (3.1)	
No	1002 (97.9)	784 (98.1)	218 (96.9)		789 (97.9)	213 (97.7)		530 (98.7)	472 (96.9)	
Probiotics n (%)				0.018			0.239			0.032
Yes	37 (3.6)	23 (2.9)	14 (6.2)		32 (4.0)	5 (2.3)		13 (2.4)	24 (4.9)	
No	987 (96.4)	776 (97.1)	211 (93.8)		774 (96.0)	213 (97.7)		524 (97.6)	463 (95.1)	
TDF (g/day), Mean (SD)	13.7 (5.6)	14.0 (5.7)	12.6 (5.3)	< 0.001	13.5 (5.6)	14.1 (5.6)	0.179	13.7 (5.9)	13.6 (5.3)	0.886

Data were expressed as mean (SD) or number (%).

Means for continuous data (age and TDF (total dietary fiber)) were compared by Welch’s t-test and proportions for categorical data (the other variables) were compared by chi-square test.

Of the 1024 participants in the analysis, 799 (78.0%) responded with having daily defecation, whereas 225 (22.0%) responded with not having daily defecation. In the following discussion, we denote the group with daily defecation as the “with defecation” (WD) group and the group without daily defecation as the “without defecation” (WOD) group. Compared with the WD group, the WOD group had a significantly higher percentage of women (57.9% vs. 76.0%; *p* < 0.001) and significantly lower total dietary fiber intake (14.0 g/day vs. 12.6 g/day; *p* < 0.001). Furthermore, compared with the WD group, the WOD group had a significantly higher percentage of participants with a history of probiotic medication use (2.9% vs. 6.2%; *p* = 0.018). Among items regarding the frequency of vinegar-based dish intake, significant differences were observed for “sour main dishes” (*p* = 0.007), “sunomono” (*p* = 0.020), and “salad with sour dressing” (*p* = 0.040); in contrast, no significant difference was noted for “pickles” (*p* = 0.270). For all items, the percentage of participants with vinegar-based dish intake habits in the WD group was higher than that in the WOD group.

Of the 1024 participants in the analysis, 806 (78.7%) reported the time required for defecation as less than 5 min, whereas 218 (21.3%) cited it as 5 min or more. In the subsequent discussion, we denote the group with less than 5 min required for defecation as the LM group and the group with 5 min or more required for defecation as the MM group. The percentage of women in the LM group was significantly higher than that in the MM group (65.3% vs. 49.5%; *p* < 0.001). Interestingly, for the total dietary fiber intake, no significant difference in the time required for defecation was observed (*p* = 0.179). No significant differences were found for “sour main dishes” (*p* = 0.562), “sunomono” (*p* = 0.059), “pickles” (*p* = 0.894), and “salad with sour dressing” (*p* = 0.909).

Out of 1024 participants in the analysis, 537 (52.4%) reported no straining during defecation, whereas 487 (47.6%) cited straining during defecation. The group without straining had a significantly lower age (68 vs. 69; *p* = 0.032) and a significantly lower percentage of participants with a history of probiotic medication use (2.4% vs. 4.9%; *p* = 0.032) than the group with straining. For other items, no significant differences were observed.

### Logistic regression analysis

For a detailed analysis of the association between the frequency of vinegar-based dish intake and defecation status, a logistic regression analysis incorporating the covariates in Tables 3, 4 and 5 was conducted. For the questionnaire item on the frequency of vinegar-based dish intake, the analysis was performed by categorizing the data and labeling the participants with an intake habit of 1 time or more/week as “Yes” and those with no intake habit as “No.”

**Table 3:** Association between daily defecation and intake habit of vinegar-based dishes.

Dependent variable: daily defecation (Yes)						
Independent variable	Model 1			Model 2		
	OR	95% CI	*p*	OR	95% CI	*p*
Sour main dishes (Yes)	1.50**	1.11–2.03	0.008	1.38*	1.02–1.88	0.039
Age (years)	0.99	0.47–2.10	0.979	0.86	0.39–1.91	0.709
Sex (Female)	0.44***	0.31–0.61	< 0.001	0.45***	0.32–0.63	< 0.001
Stomachics (Yes)	–	–	–	0.72	0.42–1.23	0.229
Laxatives (Yes)	–	–	–	0.67	0.26–1.71	0.400
Probiotics (Yes)	–	–	–	0.48*	0.24–0.97	0.042
Total dietary fiber (g/day)	–	–	–	5.28**	1.73–16.11	0.004
Sunomono	1.71**	1.19–2.44	0.004	1.49*	1.03–2.17	0.035
Age	0.87	0.41–1.88	0.733	0.80	0.36–1.80	0.595
Sex	0.41***	0.29–0.58	< 0.001	0.43***	0.30–0.61	< 0.001
Stomachics	–	–	–	0.71	0.42–1.22	0.221
Laxatives	–	–	–	0.67	0.26–1.72	0.406
Probiotics	–	–	–	0.47*	0.23–0.96	0.038
Total dietary fiber	–	–	–	4.71**	1.52–14.64	0.007
Pickles	1.24	0.91–1.69	0.180	1.08	0.79–1.50	0.622
Age	0.96	0.44–2.08	0.919	0.87	0.39–1.97	0.746
Sex	0.43***	0.31–0.60	< 0.001	0.45***	0.32–0.63	< 0.001
Stomachics	–	–	–	0.72	0.42–1.23	0.232
Laxatives	–	–	–	0.66	0.26–1.67	0.376
Probiotics	–	–	–	0.47*	0.23–0.95	0.035
Total dietary fiber	–	–	–	5.88**	1.88–18.39	0.002
Salad with sour dressing	1.53*	1.09–2.13	0.013	1.41*	1.00–1.98	0.049
Age	1.05	0.50–2.23	0.893	0.91	0.41–2.02	0.820
Sex	0.42***	0.30–0.59	< 0.001	0.43***	0.31–0.61	< 0.001
Stomachics	–	–	–	0.71	0.41–1.21	0.207
Laxatives	–	–	–	0.66	0.26–1.68	0.384
Probiotics	–	–	–	0.46*	0.23–0.94	0.033
Total dietary fiber	–	–	–	5.21**	1.70–15.97	0.004

*: *p* < 0.05, **: *p* < 0.01, ***: *p* < 0.001.

*OR* odds ratio, *CI* confidence interval

Model 1: adjusted for age and sex; Model 2: adjusted for age, sex, stomachics, laxatives, probiotics, and total dietary fiber.

**Table 4:** Association between defecation time and intake habit of vinegar-based dishes.

Dependent variable: defecation time less than 5 min (Yes)						
Independent variable	Model 1			Model 2		
	OR	95% CI	*p*	OR	95% CI	*p*
Sour main dishes (Yes)	1.12	0.83–1.52	0.463	1.16	0.85–1.58	0.352
Age (years)	0.70	0.33–1.49	0.353	0.76	0.34–1.70	0.499
Sex (Female)	1.94***	1.43–2.62	< 0.001	1.93***	1.42–2.62	< 0.001
Stomachics (Yes)	–	–	–	0.96	0.52–1.75	0.888
Laxatives (Yes)	–	–	–	0.83	0.30–2.34	0.729
Probiotics (Yes)	–	–	–	1.86	0.70–4.96	0.212
Total dietary fiber (g/day)	–	–	–	0.55	0.21–1.48	0.240
Sunomono	1.35	0.94–1.94	0.100	1.44	1.00–2.09	0.053
Age	0.64	0.29–1.38	0.252	0.70	0.31–1.59	0.399
Sex	1.89***	1.39–2.56	< 0.001	1.86***	1.37–2.53	< 0.001
Stomachics	–	–	–	0.95	0.52–1.74	0.869
Laxatives	–	–	–	0.83	0.30–2.33	0.722
Probiotics	–	–	–	1.87	0.70–4.99	0.210
Total dietary fiber	–	–	–	0.48	0.18–1.30	0.149
Pickles	1.03	0.75–1.41	0.863	1.08	0.78–1.50	0.636
Age	0.70	0.32–1.55	0.383	0.74	0.32–1.70	0.479
Sex	1.93***	1.42–2.61	< 0.001	1.92***	1.41–2.60	< 0.001
Stomachics	–	–	–	0.96	0.53–1.75	0.899
Laxatives	–	–	–	0.83	0.29–2.33	0.720
Probiotics	–	–	–	1.86	0.70–4.94	0.216
Total dietary fiber	–	–	–	0.56	0.21–1.52	0.256
Salad with sour dressing	0.97	0.68–1.37	0.859	1.00	0.70–1.42	0.987
Age	0.72	0.34–1.54	0.396	0.77	0.35–1.74	0.536
Sex	1.93***	1.43–2.62	< 0.001	1.92***	1.41–2.61	< 0.001
Stomachics	–	–	–	0.96	0.53–1.75	0.893
Laxatives	–	–	–	0.83	0.30–2.34	0.727
Probiotics	–	–	–	1.84	0.69–4.89	0.222
Total dietary fiber	–	–	–	0.59	0.22–1.59	0.298

***: *p* < 0.001.

*OR* odds ratio, *CI* confidence interval

Model 1: adjusted for age and sex; Model 2: adjusted for age, sex, stomachics, laxatives, probiotics, and total dietary fiber.

**Table 5:** Association between straining and intake habit of vinegar-based dishes.

Dependent variable: straining during defecation (No)						
Independent variable	Model 1			Model 2		
	OR	95% CI	*p*	OR	95% CI	*p*
Sour main dishes (Yes)	0.94	0.73–1.20	0.607	0.91	0.71–1.17	0.466
Age (years)	0.51*	0.27–0.95	0.034	0.55	0.28–1.05	0.072
Sex (Female)	1.13	0.88–1.46	0.334	1.15	0.89–1.49	0.271
Stomachics (Yes)	–	–	–	0.88	0.55–1.43	0.614
Laxatives (Yes)	–	–	–	0.49	0.20–1.24	0.132
Probiotics (Yes)	–	–	–	0.53	0.26–1.07	0.075
Total dietary fiber (g/day)	–	–	–	1.43	0.62–3.30	0.397
Sunomono	1.15	0.84–1.56	0.385	1.11	0.81–1.52	0.508
Age	0.48*	0.25–0.89	0.021	0.52	0.27–1.01	0.055
Sex	1.12	0.87–1.45	0.373	1.15	0.89–1.48	0.301
Stomachics	–	–	–	0.88	0.54–1.43	0.608
Laxatives	–	–	–	0.50	0.20–1.24	0.135
Probiotics	–	–	–	0.54	0.27–1.08	0.081
Total dietary fiber	–	–	–	1.29	0.55–3.00	0.556
Pickles	1.04	0.80–1.34	0.777	1.01	0.77–1.31	0.953
Age	0.49*	0.26–0.93	0.029	0.54	0.27–1.05	0.068
Sex	1.13	0.88–1.46	0.334	1.16	0.89–1.49	0.269
Stomachics	–	–	–	0.88	0.55–1.43	0.615
Laxatives	–	–	–	0.49	0.20–1.24	0.134
Probiotics	–	–	–	0.54	0.27–1.08	0.079
Total dietary fiber	–	–	–	1.36	0.58–3.18	0.474
Salad with sour dressing	1.10	0.83–1.46	0.502	1.09	0.82–1.45	0.565
Age	0.50*	0.27–0.92	0.026	0.54	0.28–1.04	0.064
Sex	1.13	0.87–1.45	0.356	1.15	0.89–1.49	0.290
Stomachics	–	–	–	0.88	0.54–1.43	0.605
Laxatives	–	–	–	0.49	0.20–1.24	0.133
Probiotics	–	–	–	0.53	0.27–1.07	0.079
Total dietary fiber	–	–	–	1.32	0.57–3.04	0.515

*: *p* < 0.05.

*OR* odds ratio, *CI* confidence interval

Model 1: adjusted for age and sex; Model 2: adjusted for age, sex, stomachics, laxatives, probiotics, and total dietary fiber.

The association between defecation frequency and vinegar-based dish intake habit was examined using logistic regression by setting participants with daily defecation as 1 (Table 3). In Model 1, with age and sex as covariates, a significant positive association was observed for defecation frequency with “sour main dishes” (odds ratio [OR]: 1.50; *p* = 0.008), “sunomono” (OR: 1.71; *p* = 0.004), and “salad with sour dressing” (OR: 1.53; *p* = 0.013). For all items, women tended to have a lower defecation frequency. In Model 2, incorporating age, sex, total dietary fiber intake, and medication history as covariates, a significant positive association was observed for defecation frequency with “sour main dishes” (OR: 1.38; *p* = 0.039), “sunomono” (OR: 1.49; *p* = 0.035), and “salad with sour dressing” (OR: 1.41; *p* = 0.049). Among the covariates, a significant positive association between defecation frequency and total dietary fiber intake was noted, whereas a significant negative association between sex (Female) and probiotic use was found.

The association between defecation time and vinegar-based dish intake habit was analyzed using logistic regression by setting participants with a defecation time of less than 5 min as 1 (Table 4). In Model 1, no significant difference was observed for any of the vinegar-based dish intake habits; however, the defecation time was significantly shorter in women. In the analysis with Model 2, a significant association was also observed only with sex.

The association between straining during defecation and vinegar-based dish intake habit was analyzed using logistic regression by setting participants with no straining during defecation as 1 (Table 5). In Model 1, no significant difference was observed for any of the vinegar-based dish intake habits; however, straining significantly increased with age. No significant association was observed for any item in Model 2.

## Discussion

This cross-sectional study revealed that the habit of eating some vinegar-based dishes was associated with defecation status. To the best of our knowledge, this is the first study to report on the association between the frequency of vinegar-based dish intake and the defecation status.

Our analysis identified a strong correlation between dietary fiber intake and defecation frequency. Dietary fiber is broadly classified into insoluble and soluble according to its water-solubility, and each affects the gut environment with different mechanisms^32^. Most insoluble fibers, such as cellulose and hemicellulose, are not digested by gut bacteria when they reach the large intestine or are only digested slowly; hence, they likely help increase stool volume. In contrast, water-soluble dietary fiber does not contribute to an increase in stool volume; however, because it is fermented in the large intestine, short-chain fatty acids are produced as metabolites, improving the gut environment. The intake of wakame, a type of algae, changes the gut environment and increases the defecation frequency^33^. Moreover, dietary fiber contributes to an improvement in defecation^16,34,35^.

The items found to have a significant association with defecation frequency, such as “sunomono” and “salad with sour dressing,” often contain seaweed and vegetables, which are rich in dietary fiber. Interestingly, even the analysis using the logistic regression model incorporating total dietary fiber intake as a covariate revealed an association between the frequency of vinegar-based dish intake and defecation frequency. Although acetic acid taken orally is absorbed in the stomach and small intestine, with most of it not reaching the large intestine^36,37^, acetic acid has been reported to improve defecation in mice when constipated mice were made to ingest acetylated starch that delivered acetic acid directly to the large intestine^22^. This may be because acetic acid altered the gut microbiota, reducing the reabsorption of water by shortening the intestinal transit time, thereby improving defecation due to the high water content in the stool. Moreover, high acetic acid concentrations in the large intestine promote serotonin secretion from enterochromaffin cells in the large intestine^23,24^, and the arrival of acetic acid in the large intestine likely causes serotonin-mediated stimulation of peristalsis.

Based on the significant association between the frequency of vinegar-based dish intake and defecation frequency, ingesting vinegar along with a matrix of foods as meals likely enables ingested acetic acid to reach the large intestine, producing such outcomes. Moreover, through acid treatment, some insoluble dietary fibers are changed to water-soluble dietary fiber, which is easily fermented in the intestine^38^. In dishes such as “sunomono,” in which foods rich in dietary fiber are subjected to acidic conditions for a long time, the amount of water-soluble dietary fiber increases, which likely affects the gut microbiota.

No association between age and defecation frequency was found, which is inconsistent with the findings of many previous studies reporting that the risk of constipation increases with age^5,7,8^. This is likely due to those older than 40 years of age participating in the health check survey, resulting in no data on younger people. In the future, intervention trials enrolling younger people should be conducted to investigate whether vinegar can improve defecation in a wide range of age groups.

Similar to previous studies^5,39,40^, we found that women had less frequent defecation than men. While the factors affecting women being more prone to constipation are not fully understood, fluctuations in the balance of female hormones such as progesterone and estrogen likely affect constipation^41,42^. Because the hormonal balance in women changes between pre- and post-menopause, it is also important to verify how the effect of defecation improvement changes between younger and older women.

Although previous studies on the time of defecation are scarce, in addition to defecation frequency, a sensation of incomplete evacuation and the use of manual maneuvers to facilitate defecation are defined as criteria in Rome IV, which defines the diagnostic criteria for functional constipation^43^. Additionally, the ability to defecate smoothly in a short time is likely to be an important indicator of constipation. Interestingly, no significant association was found between the time required for defecation and the total intake of dietary fiber. Moreover, while there was no significant association between the time required for defecation and the habit of eating vinegar-based dishes with respect to any of the items, participants with a habit of “sunomono” intake showed a tendency for shorter defecation. Even for people who do not experience any change in their defecations with dietary fiber intake, “sunomono” intake may improve the defecation status through some other mechanism than dietary fiber. In contrast, even though women tend to have less frequent defecations, we found that the time required for defecation was significantly shorter in women. Women have lower stool volume than men^44^; hence, this may lead to a shorter defecation time.

Unfortunately, there was no significant association between straining and the habit of eating vinegar-based dishes. Given that straining tends to significantly increase with age, it may be necessary for older people’s defecation to strain irrespective of the frequency and duration of defecation.

Medication history of gastrointestinal supplements had a negative correlation with the frequency and time required for defecation. Normally, primary care for improving defecation involves the improvement of eating and lifestyle habits, and treatment with gastrointestinal supplements is selected only if there is no improvement^12^. Our results showing a negative association between medication history and defecation status may not have been from gastrointestinal supplements worsening defecation; rather, it was likely that patients with poor defecation were using gastrointestinal supplements, resulting in causal reversal.

As for the habit of eating “pickles,” no association with defecation status was observed. This may be because the amount per serving is smaller than one of the other survey items in the case of Japanese people. This study is not an interventional trial; hence, we cannot accurately discuss the amount of intake or its effects. We propose that further research should be conducted to elucidate the mechanism of defecation improvement due to vinegar-based dishes, the required amount of acetic acid, and the relationship between acetic acid and dietary fiber.

This study had a few limitations. First, the amount of vinegar intake was not known. Because of the lack of information on the method and amount of intake, we could not ascertain the exact amount of vinegar intake. Second, unknown covariates were likely present. Factors other than sex, age, total dietary fiber intake, and medication history likely affect defecation. Third, owing to the cross-sectional study design, we were unable to prove a direct causal relationship between vinegar intake and improved defecation frequency. However, it is interesting to note that people with the habit of ingesting vinegar through meals tend to have better defecation. In the future, if we can elucidate a causal relationship between vinegar intake and improved defecation through an intervention study comparing a vinegar-intake group and a non-vinegar-intake group, we may be able to provide people a new option for constipation relief and contribute to their health.

Nevertheless, our study had several strengths and novelties. This cross-sectional study was the first to investigate the relationship between the habit of eating vinegar-based dishes and defecation status, considering various confounding factors. We emphasized that these relationships were independent of the intake of dietary fiber, which is well known as a nutrition fact to improve defecation status.

In conclusion, this study elucidated that the habit of eating some vinegar-based dishes, such as “sour main dishes,” “sunomono,” and “salad with sour dressing,” at least 1 time/week was associated with defecation frequency. Hence, the habit of consuming vinegar through such forms of food may be a novel approach for improving defecation status.

## Data Availability

The data presented in this study are available on request from the corresponding author. The data are not publicly available due to privacy and ethical restrictions.
